# Military Personals

**Published:** 1918-06

**Authors:** 


### MILITARY PERSONALS

To Fort Oglethorpe, Ga., Lieuts. Lee M. Sachs, Haworth R. Traver, Buffalo; Anthony Bondi, Harold L. St. John, Rochester; George M. Opperman, Michael J. McMahan, Frank N. Potts, Chas. Simon, Buffalo; Floyd H. Jones, Elmira;

Salavator C. Loiacono, Buffalo; W. W. Millas, Rome; Robert C. Scott, Syracuse; Frank H. Snyder, Geneva; Capts. Wallace Aubry, Cohoes; Emerson W. Ayars, Alfred.

To Port McHenry, Md., Lieuts. Brooks W. McCuen, Syracuse; H. W. Johnson, Gowanda.

To New Orleans, La., Lieut. Frank E. Fox, Fulton.

To New York City, Lieuts. Frank C. Walz, Buffalo; James F. Munson, Sonyea.

To Philadelphia, Pa., Lieuts. Clarence B. Gould, Batavia; J. Y. Cohen, Buffalo.

To Rockefeller Institute, Lieuts. James C. Sullivan, Buffalo; Ralph F. Gregerious, Corning; Joseph A. Newicki, Buffalo; Brewster C. Deust, Syracuse.

Honorably discharged for physical disability, Lieuts. Ward W. Millias, Rome; W. E. Barren, Addison.

Letter directing Lieut. Harold E. Shaver, Sherman, N. Y., to Hoboken, revoked.

To Camp A. A. Humphreys, Accotink, Va., Major Arthur S. Moore, Middletown.

To Camp Gordon, Lieuts. Frank Kruse, Chas. C. Panzarella, Buffalo; Henry M. Spofford, Batavia; P. H. Buckley, Buffalo.

To Camp McClellan, Lieuts. Walter L. Weeden, Chas. J. Hunt, Clifton Springs.

To Camp Wadsworth, Lieut. Lee A. Hadley, Syracuse.

To Fort Benjamin Harrison, Capt. Chas. H. Erway, Elmira.

To Hoboken, N. J., Lieut. Harold E. Shaver, Sherman; Capt. John R. Bradley, Rochester.

To Camp MacArthur, Capt. R. R. McCully, Auburn.

To Camp Meade, Lieut. Harry E. Wheelock, Jamestown.

To Camp Wheeler, Major Harvey Gaylord, Buffalo.

To Camp Dix, Lieuts. Frank Walz, Buffalo; L. E. McCanna, Elmira; Elias C. Fischbein, Sonyea; Capt. W. K. Willoughby, Auburn.

To Camp Forest, Ga., Capt. Chas. E. Erway, Elmira; Lieut. Raymond B. Morris, Olean.

To Camp Greene, Capt. R. B. Bontecou, Ithaca.

To Fort McDowell, Capt. Kent E. Williams, Rome.

To Mineola, L. I., Lieut. W. B. White, Jamestown.

To Army Med. School, Lieuts. D. A. MacDuffie, Olean; R. E. Elliott, Rochester.

To Camp Dodge, Capt. Kent E. Williams, Rome.

To Camp Hancock, Lieut. W. E. Diefenbach, Nunda.

Dr. William Stanton of Webster received the commission of Captain last June. He writes that in April he was made a Major. He is stationed at Fort Slocum, N. Y., where, since the first of Dec. he has examined personally the nose, ears and throat of over 27,000 men. He has a son serving with the Marines in France and one serving as a cadet in the Aviation Corps.

To Boston, Mass., Lieut. Herbert I. Kallet, Syracuse.

To Camp Colt, Gettysburg, Pa., Lieuts. Frank A. Walder, Lockport; Brooks W. McCuen, Syracuse.

To Camp Grant, Lieut. Norman L. Sheehe, Dunkirk.

To Camp Upton, Lieuts. Adelbert C. Abbott, Syracuse; Ira W. Livermore, Gowanda.

Health Commissioner Fronczak of Buffalo has been drafted by the government for service in the medical corps of the national army. He has been commissioned a Major. Dr. Franklin Gram will serve as Commissioner during Dr. Fronczak's absence from town.

Dr. Frank Talbot of Niagara Falls has been promoted from Lieut.-Surg. of the U. S. Med. Corps. He is now stationed at Camp Devens, Ayer, Mass.

Dr. Harry Maldiner of N. Tonawanda, a Lieut. with the medical staff of the American forces in France writes that his Christmas packages came late in March and were in good condition.

Dr. David Stoddard Dooman and Dr. Eucarpio Pisani of Buffalo have received commissions as lieutenants in the M. R. C.

